# Antigenic Analysis of Monoclonal Antibodies against Different Epitopes of σB Protein of Avian Reovirus

**DOI:** 10.1371/journal.pone.0081533

**Published:** 2013-11-27

**Authors:** Chun-hong Yin, Li-ting Qin, Mei-yu Sun, Yu-long Gao, Xiao-le Qi, Hong-lei Gao, Yong-qiang Wang, Xiao-mei Wang

**Affiliations:** Division of Avian Infectious Diseases, State Key Laboratory of Veterinary Biotechnology, Harbin Veterinary Research Institute, Chinese Academy of Agricultural Sciences, Harbin, P. R. China; University of Alabama at Birmingham, United States of America

## Abstract

**Background:**

Avian reovirus (ARV) causes arthritis, tenosynovitis, runting-stunting syndrome (RSS), malabsorption syndrome (MAS) and immunosuppression in chickens. σB is one of the major structural proteins of ARV, which is able to induce group-specific antibodies against the virus.

**Methods and Results:**

The present study described the identification of two linear B-cell epitopes in ARV σB through expressing a set of partially overlapping and consecutive truncated peptides spanning σB screened with two monoclonal antibodies (mAbs) 1F4 and 1H3-1.The data indicated that ^21^KTPACW^26^ (epitope A) and ^32^WDTVTFH^38^ (epitope B) were minimal determinants of the linear B cell epitopes. Antibodies present in the serum of ARV-positive chickens recognized the minimal linear epitopes in Western blot analyses. By sequence alignment analysis, we determined that the epitopes A and B were not conserved among ARV, duck reovirus (DRV) and turkey reovirus (TRV) strains. Western blot assays, confirmed that epitopes A and B were ARV-specific epitopes, and they could not react with the corresponding peptides of DRV and TRV.

**Conclusions and Significance:**

We identified ^21^KTPACW^26^ and ^32^WDTVTFH^38^ as σB -specific epitopes recognized by mAbs 1F4 and 1H3-1, respectively. The results in this study may have potential applications in development of diagnostic techniques and epitope-based marker vaccines against ARV groups.

## Introduction

Avian reovirus (ARV) belongs to the genus *Orthoreovirus* in the family *Reoviridae*. It was first isolated from chickens in 1954 [1]. Since then, many variants with broad antigenic diversity have been isolated worldwide [2-6]. ARV has been reported to be associated with a number of disease syndromes, including arthritis/tenosynovitis, enteric disease (runting-stunting syndrome (RSS), malabsorption syndrome (MAS)), respiratory disease and some other syndromes [7-9]. Chickens are most susceptible to this disease at a young age, and protection of offspring is conferred by antibodies transferred from vaccinated maternal flocks [10,11]. The S1133 virus is the standard challenge virus for VA and is in most VA vaccines around the world. It was isolated from sick chickens around 50 years ago [12].

The ARV genome consists of 10 fragments of double-stranded RNA of 1 to 4 kb that are divided into large (L), medium (M) and small (S) according to their size [13,14]. There are at least 10 structural proteins (λA, λB, λC, μA, μB, μBC, μBN, σC, σA, and σB) and 4 nonstructural proteins (μNS, P10, P17, and σNS) encoded by these genes. σB, a major outer capsid protein of ARV, is structurally related to the σ3 protein of mammalian reovirus (MRV) and the σB protein of DRV, which suggests it is a functional protein. The σB protein of ARV comprises 367 amino acids and is able to induce group-specific neutralizing antibodies [15,16]. The σB proteins of the ARV strains contain conserved immunogenic regions, suggesting that the σB protein can potentially be used as a target to raise pan-ARV σB mAbs for the detection of a broad spectrum of avian reoviruses. Among these proteins, σB, σC and μB/μBC can induce neutralizing antibody against ARV [17-19]. Efforts have been made to map the epitopes of σ-class proteins, including σA, σC and σNS [20-23]. However, σB protein epitope antigenicity information remains unclear.

A detailed analysis of epitopes is important for the understanding of immunological events, the development of epitope-based marker vaccines and the development of diagnostic tools for various diseases [24,25]. In this study, two monoclonal antibodies (mAbs) against σB protein were developed. Partially overlapping and consecutive truncated peptides spanning the σB protein were screened by mAbs to identify the locations of mAbs epitopes.

## Materials and Methods

### Ethics Statement

Care of laboratory animals and animal experimentation were performed in accordance with animal ethics guidelines and approved protocols. All animal studies were approved by the Animal Ethics Committee of Harbin Veterinary Research Institute of the Chinese Academy of Agricultural Sciences (SYXK (H) 2006-032).

### Viruses and Cells

ARV (S1133strain) isolated from an America chicken flock was cultured in chicken embryo fibroblasts (CEFs) that were prepared with 10-day-old chicken embryo. The CEF cells were grown in Dulbecco’s modified Eagle’s medium (DMEM) supplemented with 5% fetal bovine serum.

### MAbs Production and Characterization

The full-length σB coding sequence was amplified using the primers ARV σB-F (5’- GTTC*GAATTC*ATGGAGGTACGTGTG-3’) and ARV σB-R (5’-GAC*GTCGAC*TTACCAACCACACTT-3’) and then cloned into a pET30a vector. Recombinant σB protein was expressed in *E. coli*BL21. Six-week-old female BALB/c mice were immunized with 100 μg of purified recombinant σB protein emulsified with Freund’s complete adjuvant (Sigma, St Louis, MO, USA). Two boosters were given with three-week intervals. Two weeks after the third immunization, the mice were intraperitoneally boosted with 100 μg antigen alone. Three days later, the spleen cells isolated from immunized mice were fused with myeloma cells, SP2/0, using 50% (wt/vol) polyethylene glycol (PEG). The hybridoma cells were screened by indirect enzyme-linked immunosorbent assay (ELISA) coated with purified σB protein or ARV particles. The hybridoma cells producing mAbs were cloned three times by the limiting dilution method. Antibody subtype identification was performed using the Pierce Rapid ELISA Mouse mAb Isotyping Kit (Thermo scientific, USA).

### Enzyme-Linked Immunosorbent Assay

Plates were coated with 100 μL/well of antigen diluted in carbonate-bicarbonate buffer (pH 9.6) and blocked with 200 μL/well of blocking buffer (PBS containing 5% skim milk). The supernatant of the hybridoma culture (100 μL/well) was added, and the plates were incubated for 1 h at 37°C. After washing three times, 100 μL of horseradish peroxidase (HRP)-conjugated goat anti-mouse immunoglobulin G (IgG, 1:5,000 dilution, Sigma, St Louis, MO, USA) was added to each well and incubated for 1 h at 37°C. The plates were washed three times and incubated with 100 μL/well of o-phenylenediamine dihydrochloride (OPD, Sigma, St Louis, MO, USA) containing 0.3% H_2_O_2_ for 15 minutes at room temperature. The reaction was stopped with 100μL/well of 2 M H_2_SO_4_ and the absorbance measured at 490 nm.

### Indirect Immunofluorescence Assay

Approximately 80% confluent CEF cells cultured in 24-well plates were infected with ARV S1133 strain at an MOI of 0.1. At 24 hours post-infection, the infected cells were fixed with ethanol absolute for 15 min. The fixed cells were incubated with mAb 1F4 and 1H3-1 or ARV-positive sera for 1 h at 37°C. After washing three times, 200 μL/well of FITC-conjugated goat anti-mouse IgG (Sigma, St Louis, MO, USA) at 1:200 dilution were added and incubated for 1 h at 37°C in dark. The cells were washed three times and then observed under a Carl Zeiss Vision microscope equipped with AXioVision Rel.4.8 software.

### Micro-Neutralization Assay

The neutralizing activities of the mAbs were determined by a micro-neutralization assay on CEF cells using ARV-S1133. The in vitro micro-neutralization assay was performed according to a modified protocol [26]. Cells infected with 200TCID_50_ virus and cells with mock infection served as positive and negative controls in the assay respectively. The assay medium for ascitic fluid and virus dilution was the complete growth medium for CEF cells. The ascitic fluid were inactivated at 56°C for 30 min and serially diluted twofold in a micro titer plate with 50 μl per well. Eight serial 2-fold dilutions (1:20 - 1:2560) of ascitic fluid in a volume of 50 μl were loaded into the plate wells, followed by the addition of 200 TCID50 virus S1133. The plates were incubated at 37°C for 1 h. Then the mixtures were added in the 96 well plate with 5×10^3^ CEF cells. The plates were incubated at 37°C for 48 h. Cells in the 96 well plate were then stained with the dye crystal violet (5% in PBS), which stains only living cells here. If a ascitic fluid contains antibody that blocks viral infection, most of the cells will survive and present violet color; if the virus can’t be blocked by the ascitic fluid, cells will be infected, round up and detach from the cell culture plate, thus no violet staining is visible for infected.

### Expression of Overlapping or Consecutive Truncated σB Protein Fragments

To localize the epitopes A and B on σB protein, a series of overlapping peptide fragments of the σB of ARV S1133 strain were expressed. At first, ten partially overlapping nucleotide fragments spanning the σB gene were amplified and then cloned into the pET-32a or pGEX6p-1 vectors. *EcoR*I and *Sal*I restriction endonucleasesites were introduced into the corresponding primers (Table S1). The recombinant plasmids were verified by DNA sequencing and transformed into *E. coli* BL21. The recombinant overlapping proteins (named Pep 1-Pep 10) were expressed with the induction of IPTG and then screened by Western blot with mAbs.

To further map the epitopes of σB, two sets of three (named Pri 11-Pri 13) and six (named Pri 14-Pri 19) pairs of partially overlapping or consecutive truncated oligonucleotides spanning gene coding 16-35 aa and 31-50 aa of σB protein were designed, respectively. The complementary oligonucleotide pairs encoding each peptide fragment were synthesized. After direct annealing, the sticky-ends of *EcoR*I and *Sal*I restriction endonucleasesites were formed at the termini of the double-stranded oligonucleotides. Then the oligonucleotides were cloned into pGEX6p-1 and digested by the same restriction enzymes. The recombinant proteins were expressed in *E. coli* BL21 with the induction of IPTG and then screened by Western blot with mAbs. The positions of the peptides and the synthesized complementary oligonucleotide pairs are listed in Table S1.

### Precise Localization of the Epitopes

To define the epitopes precisely, seven mutants with truncations at the amino- or carboxy-termini of the motif ^21^KTPACWNAQT^30^ (Table 1) were constructed to express the GST fusions GST-AΔT, GST-AΔQT, GST-AΔAQT, GST-AΔNAQT, GST-AΔWNAQT, GST-AΔK, and GST-AΔKT representing KTPACWNAQ-, KTPACWNA-, KTPACWN-, KTPACW-, KTPAC-, -TPACWNAQT and -PACWNAQT (deletions indicated as dashes), respectively. With the same method, nine mutants with deletions at the amino- or carboxy-termini of another motif ^32^WDTVTFHVPDVIRV^45^ (Table 1) were constructed. The GST fusions, GST-BΔV, GST-BΔRV, GST-BΔIVR, GST-BΔVIVR, GST-BΔDVIRV, GSTBΔPDVIRV, GST-BΔVPDVIRV, GST-BΔHVPDVIRV and GST-BΔW, representing WDTVTFHVPDVIR-, WDTVTFHVPDVI-, WDTVTFHVPDV-, WDTVTFHVPD-, WDTVTFHVP-, WDTVTFHV-, WDTVTFH-, WDTVT-, -DTVTFHVPDVIR, respectively, were expressed in *E. coli* BL21. All the DNA segments and recombinant peptides were obtained as described in Materials and Methods. The series of GST fusions were screened by Western blot with mAb 1F4 or 1H3-1, respectively.

**Table 1:** The sequence of the truncated peptides of the motif A (^21^KTPACWNAQT^30^) and B (^32^WDTVTFHVPDVIRV^45^).

**Peptides**	**Coding motifs**	Reaction*
GST-A	KTPACWNAQT-	**+**
GST-AΔT	KTPACWNAQ-	**+**
GST-AΔQT	KTPACWNA-	**+**
GST-AΔAQT	KTPACWN-	**+**
GST-AΔNAQT	KTPACW-	**+**
GST-AΔWNAQT	KTPAC-	_
GST-AΔK	-TPACWNAQT	_
GST-AΔKT	-PACWNAQT	_
GST-B	WDTVTFHVPDVIRV-	**+**
GST-BΔV	WDTVTFHVPDVIR-	**+**
GST-BΔRV	WDTVTFHVPDVI-	**+**
GST-BΔIRV	WDTVTFHVPDV-	**+**
GST-BΔVIRV	WDTVTFHVPD-	**+**
GST-BΔDVIRV	WDTVTFHVP-	**+**
GST-BΔPDVIRV	WDTVTFHV-	**+**
GST-BΔVPDVIRV	WDTVTFH-	**+**
GST-BΔHVPDVIRV	WDTVTF	_
GST-BΔW	-DTVTFHVPDVIRV	_

Notes: * represented the peptides reactivity with mAb1F4 or 1H3-1.

+ represents the positive result.

– represents the negative result.

### SDS-PAGE and Western Blot

Samples were mixed with a quarter volume of 5× sample loading buffer, boiled for 10min, and subjected to 15% sodium dodecyl sulfate-polyacrylamide gel electrophoresis (15% SDS–PAGE). The gels were stained with Coomassie brilliant blue staining solution or electroblotted onto nitrocellulose (NC) membranes. After being blocked with 5% skim milk dissolved in PBS, the membrane was incubated with mAbs 1F4 and 1H3-1 at 37°C for 1 h. After being washed three times with PBS, the membrane was probed with a 1:5,000 dilution of HRP-conjugated goat anti-mouse IgG (Sigma, St Louis, MO, USA) at 37°C for 1 h. Reactivity was visualized with the substrate 3, 3’-diaminobenzidine (DAB; Sigma, St Louis, MO, USA).

### Homology and Cross-Reactivity

To investigate the homology of the identified epitopes among ARV, DRV and TRV, 31 published sequences, including 18 ARV, 8 DRV and 5 TRV strains, were aligned using the DNASTAR Lasergene program (Windows version; DNASTAR Inc., Madison, WI). The GenBank accession numbers for the ARV/DRV/TRV σB-encoding genes are listed in Table S2. According to the results of the alignment, 6 mutant peptides were expressed as GST fusion proteins (designated as M1-A, M2-A, M3-A; M4-B, M5-B, M6-B, M7-B; Table 2). Then, these proteins were screened by Western blot with mAbs 1F4 and 1H3-1, respectively.

**Table 2:** The oligonucleotides and according coding motifs for homology assays.

**epitopes**	**peptides**	**The sequences of oligonucleotide**	**Coding motifs**
**A**	M1-A	**CGGTCT**CCTGCTTGCTGG	**RS**PACW
	M2-A	**CGG** **GCT**CCTGCTTGCTGG	**RA**PACW
	M3-A	**CGGACT**CCTGCT TGCTGG	**R**TPACW
**B**	M4-B	TGGGAT**ATTGAAGAG**TTTCAC	WD**IEE**FH
	M5-B	TGGGAT**AGTGATATC**TTT CAC	WD**SDI**FH
	M6-B	TGG**AATATTGAA** ACT TTTCAC	W**NIE**TFH
	M7-B	TGGGAT**GTTGAA**ACTTTT CAC	WD**VE**TFH

Notes: M (1-3)-A represents the corresponding peptides of epitope A, and M (4-7)-B represents the corresponding peptide of epitope B. The bold font letters represent the different nucleotides and amino acid residues from avian reovirus S1133.

## Results

### Production and Identification of the MAbs

**Figure 1:**
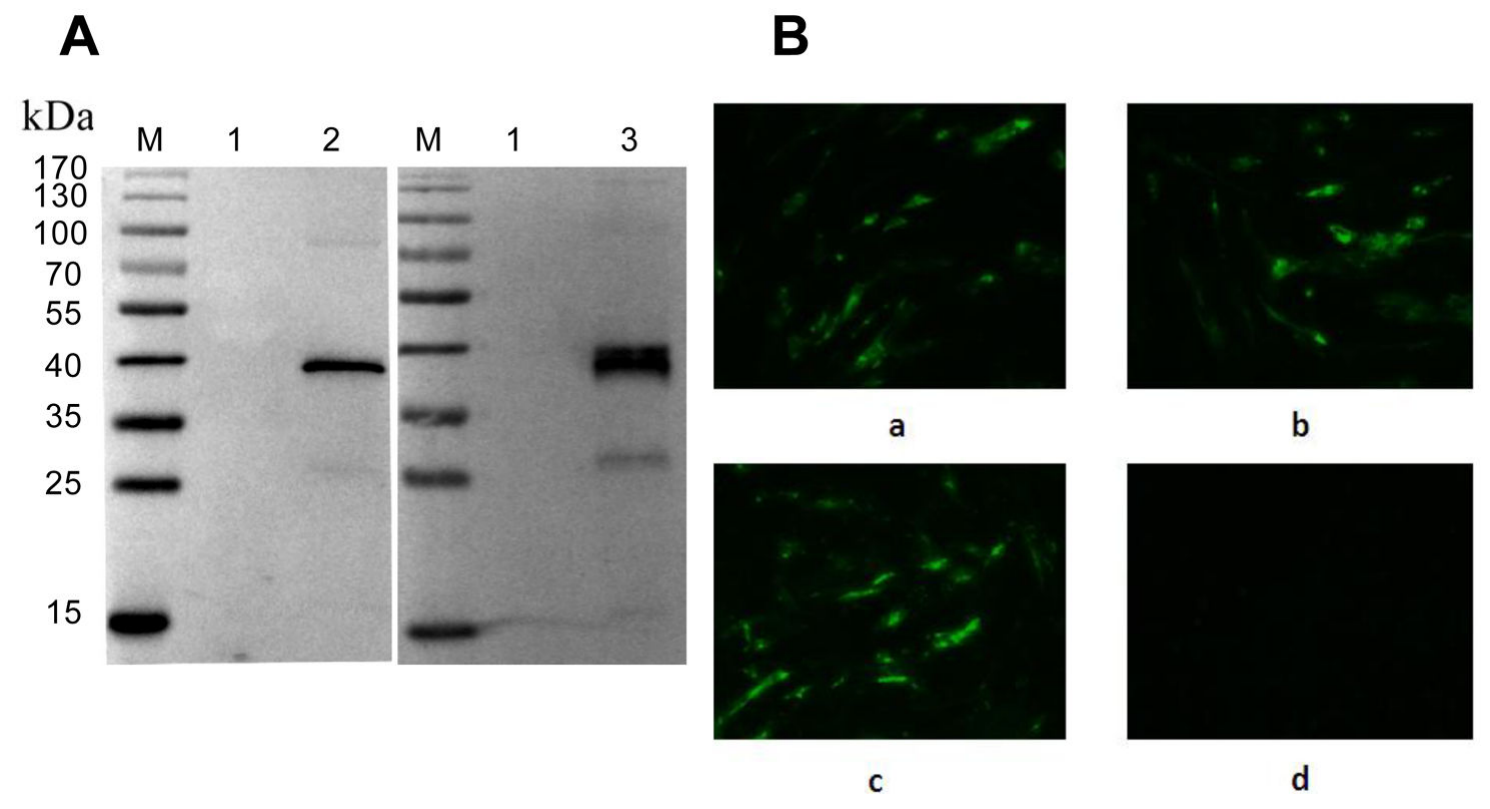
Reaction of the two MAbs (1F4 and 1H3-1) with ARV. (A) Identification of reactivity of the mAbs 1F4 and 1H3-1 with the purified avian reovirus (ARV) using Western blot assay. Lane 1: Supernatant of CEF cell culture. Lane 2 and Lane 3: purified virus particles; (B) Identification of reactivity of the mAbs1F4 and 1H3-1 with cells infected ARV using indirect immunofluorescence assay (IFA). CEF cells in 24-well plates were infected with ARV S1133 strain at a MOI of 0.1. (a) mAb 1F4, (b) mAb 1H3-1, (c) ARV infected positive serum control, (d) negative serum control.

Purified σB protein was used to immunize BALB/c mice. After cell fusion and screening, several hybridoma cell lines were generated, which produced σB reactive mAbs. Two monoclonal antibodies with designated as 4F1 and 1H3-1 were selected for strong reactivity with purified whole virus in Western blot (Figure 1A) and IFA (Figure 1B).

The neutralizing activities of the mAb 1F4 and 1H3-1 were then determined by a micro-neutralization assay on CEF cells using S1133, respectively. The results showed that the mAbs 1F4 and 1H3 couldn’t neutralize the virus (data not shown).

### Localization of the Two Epitopes

**Figure 2:**
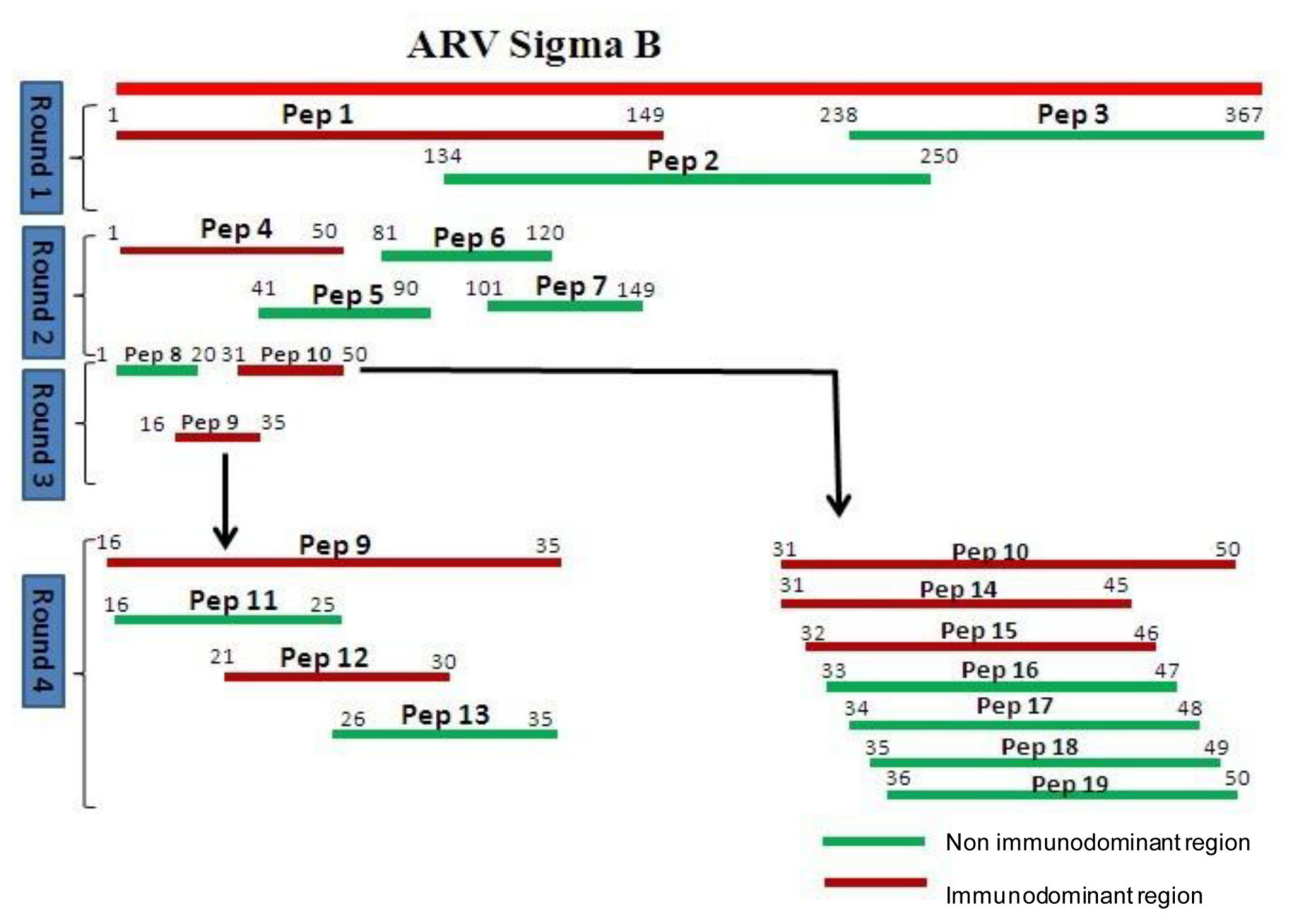
Strategy of the recombinant protein of the σB protein mapping. Round 1 to round 4 are the four rounds of expression of overlapping fragments and identification of the immunodominant region with mAbs (1F4 and 1H3-1). The red fragments are the identified immunodominant regions, and the green fragments are the non-immunodominant regions. The positions of the peptides are in accordance with the published protein sequence of the S1133 strain σB capsid protein (UniProt accession number: E7AXT8).

To map the antigenic epitope of the σB, a series of overlapping or truncated peptides fragments were expressed and used for reactivity by Western blot with mAbs (1F4 and 1H3-1). The results of the third round mapping assay showed that the two mAbs 1F4 and 1H3-1 could only recognize the peptides Pep 9 (16-35 aa) and Pep 10 (31–50 aa), respectively (Figure 2). For further analysis of the epitopes, a fourth round mapping assay was conducted and aimed at peptide Pep 9 (16-35 aa). Pep 12 (^21^KTPACWNAQT^30^) was found to react with mAb (1F4). For Pep 10(31-50 aa), nine truncated peptides were obtained and subjected to Western blot assay. The results indicate that the mAb (1H3-1) could react with both the Pep 14 (31-45 aa) and Pep 15 (32-46 aa), which indicated that the epitope of mAb (1H3-1) might be located in peptide (^32^WDTVTFHVPDVIRV^45^) (Figure 2).

### Precise Defining of the Epitopes

**Figure 3:**
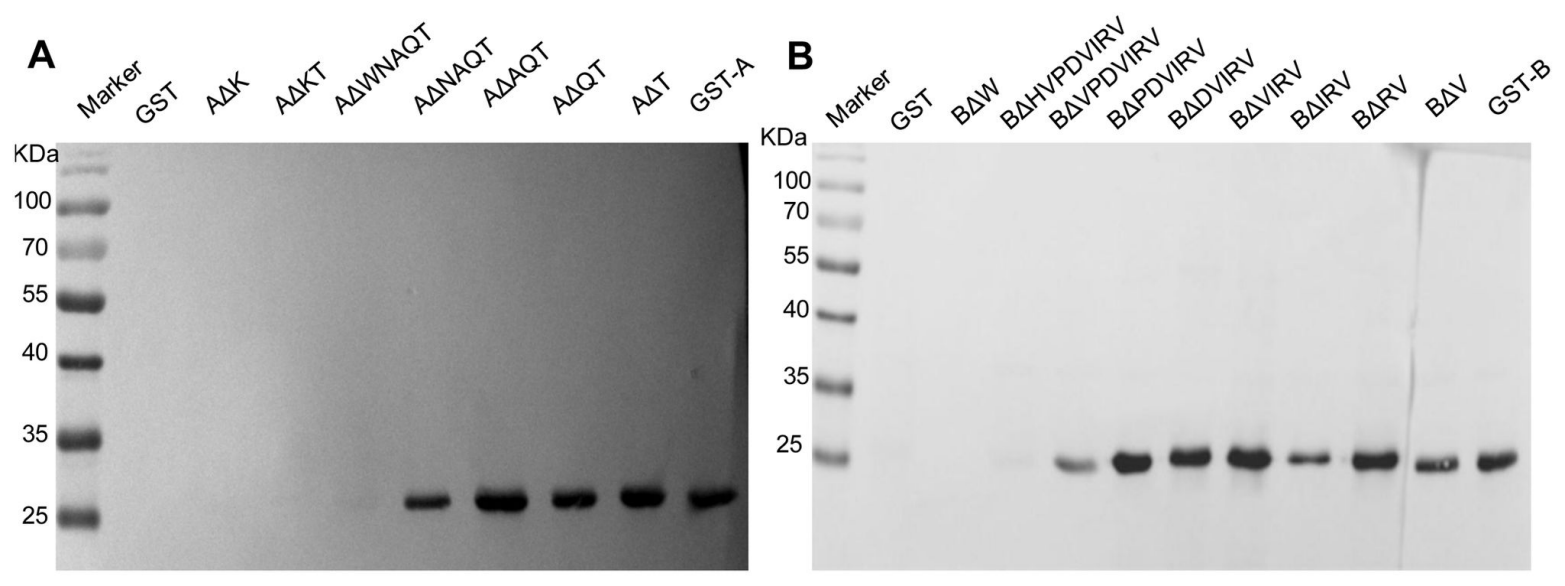
Reactivity of the GST-fusion proteins containing truncated motifs derived from the epitope ^21^KTPACWNAQT^30^ with the mAb 1F4 (A) and the epitope ^32^WDTVTFHVPDVIRV^45^ with 1H3-1 (B). Marker: PageRuler^TM^ Prestained Protein Ladder; GST: negative control; GST-A: containing the motif ^21^KTPACWNAQT^30^; AΔT: KTPACWNAQ-; AΔQT: KTPACWNA-; AΔAQT: KTPACWN-; AΔNAQT: KTPACW-; AΔWNAQT: KTPAC-; AΔK: -TPACWNAQT; AΔKT: -PACWNAQT; GST-B: containing the motif ^32^WDTVTFHVPDVIRV^45^; BΔV: WDTVTFHVPDVIR-; BΔRV: WDTVTFHVPDVI-; BΔIRV: WDTVTFHVPDV-; BΔVIRV: WDTVTFHVPD-; BΔDVIRV: WDTVTFHVP-; BΔPDVIRV: WDTVTFHV-; BΔVPDVIRV: WDTVTFH-; BΔHVPDVIRV: WDTVTF-; BΔW: -DTVTFHVPDVIRV.

The seven mutant peptides with truncations at the amino- or carboxy-termini of the motif A ^21^KTPACWNAQT^30^ were constructed to express the GST fusion proteins in *E. coli* BL21. We found that GST-AΔWNAQT, GST-AΔK and GST-AΔKT were not recognized by the mAb 1F4, and others all have binding activity with 1F4 (Figure 3A), indicating that the peptide ^21^KTPACW^26^ represented the minimal requirement for the reactivity of the epitope with 1F4.

For motif B ^32^WDTVTFHVPDVIRV^45^, nine mutants with truncations at amino- or carboxy-termini of motif were expressed in *E. coli* BL21. As the results showed, except for GST-BΔHVPDVIRV and GST-BΔW, the other motifs could all react with the mAb 1H3-1.We can conclude that the motif ^32^WDTVTFH^38^ is the minimal linear epitope (Figure 3B).

### Reactivity of Epitope Peptides with Positive Sera

**Figure 4:**
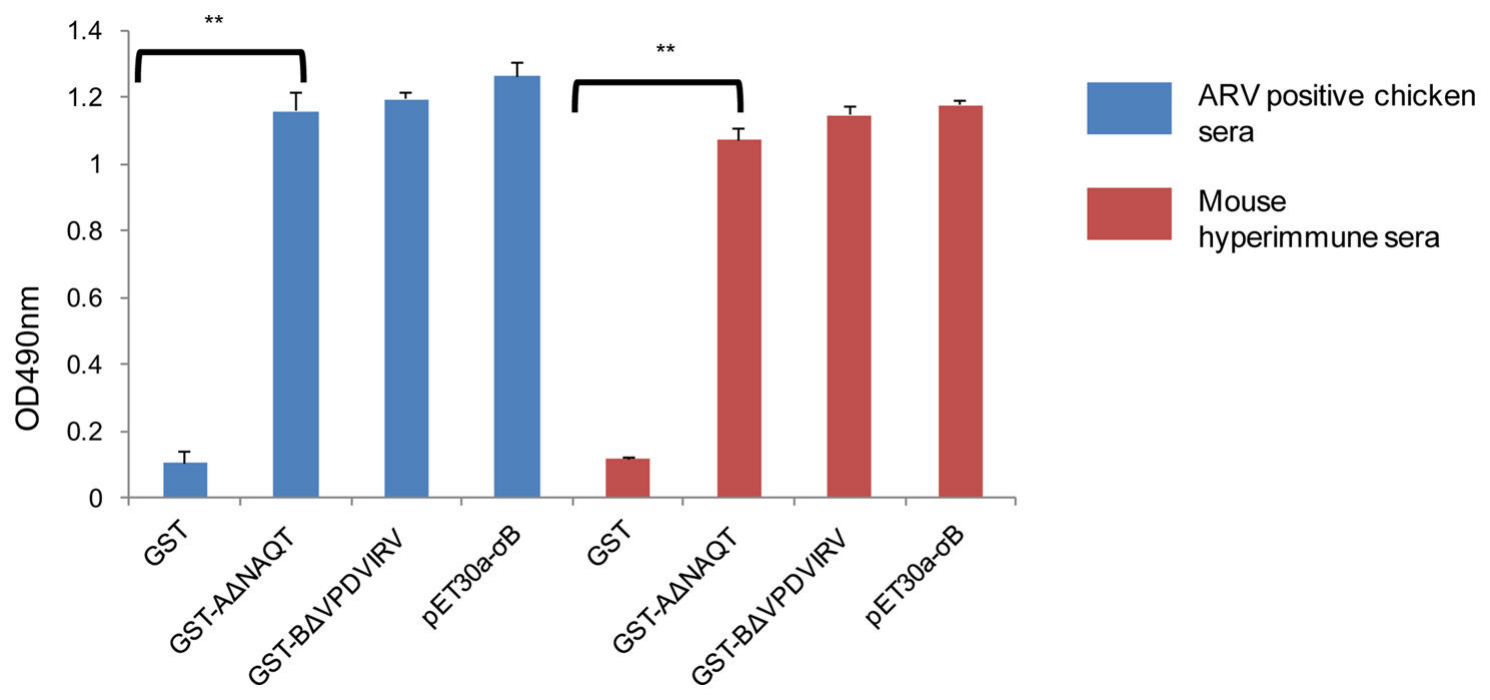
Analysis of the reactivity of two epitopes with chicken anti-ARV sera and mice anti-ARV σB sera by ELISA. Four purified proteins (GST, epitope A: GST-AΔNAQT, epitope B: GST-BΔVPDVIRV and integral σB: pET30a-σB) were used as the coated antigen (2μg/well). Vaccinated chicken sera or mouse hyperimmune sera were used to detect antibody. Three replicates of each selected coated protein were performed and statistically significant differences were determined by one-way ANOVA (**, p<0.01).

To investigate whether the epitopes could be recognized by chicken anti-ARV or mice anti-ARV σB sera, the epitope peptides GST-AΔNAQT and GST-BΔVPDVIRV were used to coat the ELISA plates (500ng/well) to examine these reactivities. The results of ELISA indicated that both the epitope peptides, GST-AΔNAQT and GST-BΔVPDVIRV, were recognized by vaccinated chicken sera or mouse hyperimmune sera (Figure 4).

### Homology and Cross-Reactivity Analysis

**Figure 5:**
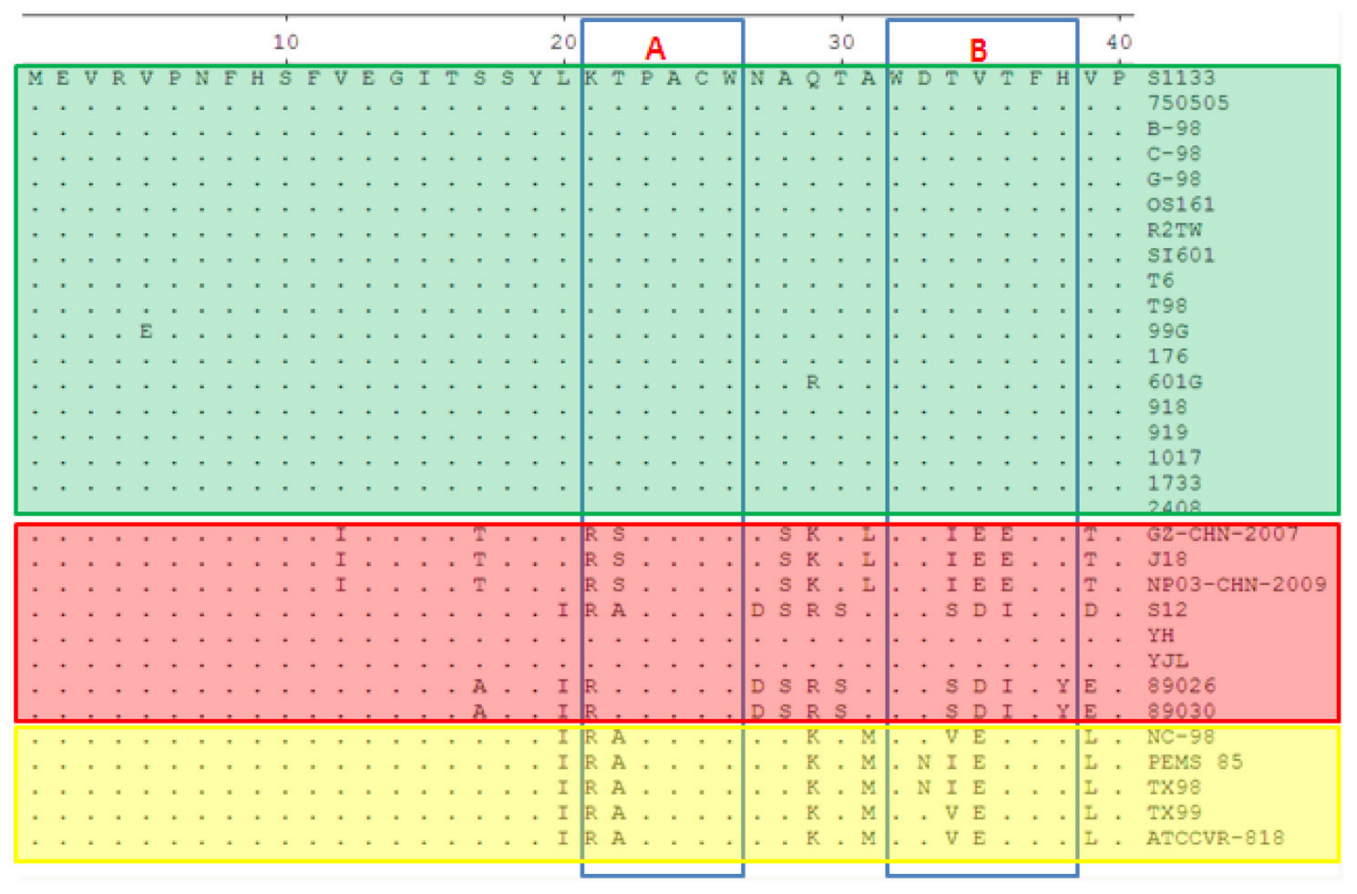
Sequence comparison of the identified epitope A and B among ARV, DRV and TRV strains available in GenBank. ARV S1133 sequence was shown on the top, and the differences were indicated. The stains in the frame in green are the ARVs. The stains in red are the DRVs, and the yellow stains represent the TRVs.

The alignment of the two epitopes, A (peptide ^21^KTPACW^26^) and epitope B (peptide ^32^WDTVTFH^38^), among ARV, DRV and TRV strains, was performed with 31 published σB protein sequences. The results revealed that both epitope A (peptide ^21^KTPACW^26^) and epitope B (peptide ^32^WDTVTFH^38^) were conserved in ARVs but not among DRV and TRV strains (Figure 5). For epitope A, two DRV strains have the same epitopes as ARV. The other strains contain a different sequence at the 21st (Lys to Arg) and 22nd (Thr to Ala or Ser) amino acid residues. In TRV, the associated peptide is ^21^RAPACW^26^. However, the peptides of the B epitope displayed more diversity in DRV and TRV (Figure 5).

**Figure 6:**
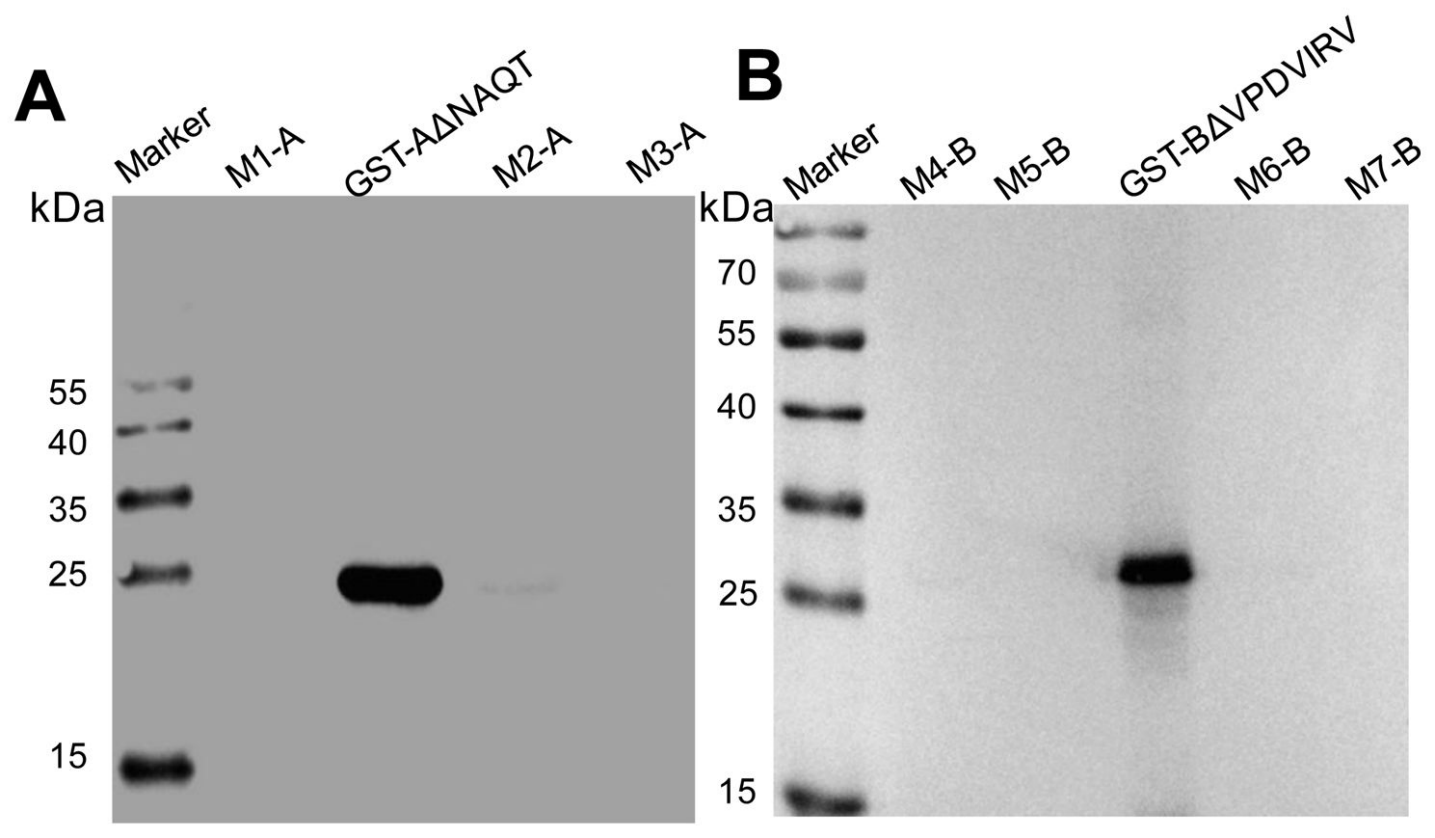
Identification of cross-reactivity of mAb 1F4 or 1H3-1 to the associated coding motif of heterologous ARV, DRV and TRV strains. Marker: PageRuler^TM^ Prestained Protein Ladder; GST-AΔNAQT containing the epitope A: KTPACW; M1-A: RSPACW; M2-A: RAPACW; M3-A: RTPACW; GST-BΔVPDVIRV containing the epitope B: WDTVTFH; M4--B: WDIEEFH; M5-B: WDSDIFH; M6-B: WNIETFH; M7-B: WDVETFH.

To test the reactivity of mAbs 4F1 and 1H3 with corresponding motifs of DRV and TRV σB protein, two classes of peptides (M1-A, M2-A, M3-A; M4-B, M5-B, M6-B, M7-B) were expressed and then screened by Western blot. The results indicated that the mAb 1F4 could not react with M1-A, M2-A and M3-A, and the other mAb 1H3-1 could not recognize the recombinant fusion proteins of M4-B, M5-B, M6-B and M7-B, which suggested that epitopes A and B are not conserved in ARVs, DRVs and TRVs (Figure 6).

## Discussion

MAbs with well-defined epitopes provide an experimental platform for studying antigen structure, developing diagnostic reagents and epitope vaccines [27-30]. The σB is one of the major outer capsid proteins of ARV and is able to induce group-specific neutralizing antibody [15,16]. The precise analysis of the epitopes in σB protein is important for understanding the mechanism of σB-mediated protection. In recent years, epitope-based marker vaccines have received more attention [31,32]. σB is antigenic and able to elicit the generation of protective antibodies. Identifying linear epitopes in σB would contribute to developing epitope-based marker vaccines.

In this study, a panel of σB-specific mAbs was produced using soluble recombinant σB expressed in *E. coli* BL21 as immunogenic antigen. Two mAbs with good reactivity with purified ARV particles and CEF infected with ARV were selected for further research. To locate the epitopes of σB protein, a set of 19 partially overlapping and consecutive truncated peptides (Pep 1-Pep 19) covering σB protein were expressed. Using the two mAbs, 1F4 and 1H3-1, the peptides of Pep 1-Pep 19 were screened by pepscan analysis. The mAb1F4 reacted with Pep 12 (21-30 aa) but did not react with the neighboring fragments Pep 11 (16–25 aa) and Pep 13(26-35 aa), which have 5 aa overlapping with Pep 12. The mAb 1H3-1 reacted with Pep 14 (31-45 aa) and Pep 15 (32-46) but did not react with the neighboring fragment Pep 16 (33–47 aa), suggesting that the amino acids 31W and 46G are not necessary for this epitope. Finally, two peptides, ^21^KTPACWNAQT^30^ and ^32^WDTVTFHVPDVIRV^45^, of ARV σB, were identified by Western blot. To further define the epitopes, a series of mutants with truncations at the amino- or carboxy-termini of the two peptides identified were constructed to express the GST fusions proteins. The recombinant fusion peptides were scanned by Western blot with mAbs. The results demonstrated that ^21^KTPACW^26^ and ^32^WDTVTFH^38^ are the minimal requirements for recognition by 1F4 and 1H3-1, respectively. It has been reported that epitopes often range from five to seven amino acids or monosaccharide residues in length and do not exceed 20 amino acids [33]. In this study, the length of the identified epitopes was six and seven amino acids, which is consistent with the previous research.

σB protein is comparatively conserved among the ARV proteins and can induce group-specific neutralizing antibody. Therefore, σB is useful for developing ARV vaccines and diagnostic reagents. In the present study, to analyze whether the two identified epitopes are in a conserved region, 31ARV, DRV and TRV strains were compared. The results indicated that epitopes A and B of avian reovirus σB are different in duck and turkey reovirus. In addition, the epitopes from the duck and turkey reoviruses could not be recognized by the mAbs 1F4 and 1H3-1, respectively. However, the epitope A and B sequences were completely conserved among ARVs. Therefore, epitope A and epitope B are genus-specific epitopes among the ARV.

The alignments of σB-deduced amino acids sequences for ARV, DRV, TRV, and Nelson Bey virus (NBV) revealed that most conserved amino acids were in the N-terminal region (1–113 aa) [34-38]. The results indicate that two epitopes identified by two mAbs both located in the N-terminal region of σB are consistent with these recent investigations. The investigator identified distinct binding motifs for zinc at the amino-terminal and dsRNA at the carboxy-terminal contained in avian σ2 protein and mammalian σ3 protein [38]. Further experiments are necessary to confirm the functional roles of these proteins.

In conclusion, two epitopes of ARV σB protein were identified using the pepscan method based on mAbs. These two epitopes are specific for the σB protein of ARV, but not for DRV and TRV. Due to these differences among ARV, DRV and TRV, differential diagnosis might be developed based on these two epitopes, and mAb1F4 and 1H3-1 could be used for discriminating ARV or DRV infection. The information provided in this study will facilitate the development of specific serological diagnosis of ARV infection and contribute to the rational design of vaccines by further the understanding of the antigenic structure of σB.

## Supporting Information

Table S1 **Primers used for peptides expression.**
(DOCX)

Table S2 **Virus sequences used for alignment and their accession numbers in GenBank.**
(DOCX)
