# Supplementary material for: Antigenic Analysis of Monoclonal Antibodies against Different Epitopes of σB Protein of Avian Reovirus
Source: PLoS One. 2013 Nov 27;8(11):e81533. doi: 10.1371/journal.pone.0081533 (PMC3842295; doi:10.1371/journal.pone.0081533)
Supplement: Table S1 — Primers used for peptides expression. (DOCX) [file pone.0081533.s001.docx]

**Table S1. Primers used for peptides expression**

| Primers | Sequences of primers and synthesized oligonucleotides | Peptides (Position in σB protein) |
| --- | --- | --- |
| Pri1 | 5-GTTC*GAATTC*ATGGAGGTACGTGTG-3 | Pep 1 (1aa-149aa) |
|  | 5-GAC*GTCGAC*TTAGTCAACCGTCTT-3 |  |
| Pri2 | 5-GTTC*GAATTC*GACATTGTAACGCGT-3 | Pep 2 (134-250) |
|  | 5-GAC*GTCGAC*TTAAAATGAATATACACC-3 |  |
| Pri3 | 5-GTTC*GAATTC*CTTGCGAATGCTGAC-3 | Pep 3 (238-368) |
|  | 5-GAC*GTCGAC*TTACCAACCACACTT-3 |  |
| Pri4 | 5-GAC*GAATTC*ATGGAGGTACGTGTGC-3 | Pep 4 (1-50) |
|  | 5-AACA*GTCGAC*TTAACAATACGCATTGCCA-3 |  |
| Pri5 | 5-TCAC*GAATTC*GATGTAATTAGAGTTG-3 | Pep 5 (41-90) |
|  | 5-TTCT*GTCGAC*TTACACATACCGGAGCAGT-3 |  |
| Pri6 | 5-GCAA*GAATTC*AGGACCGATACCCCAC-3 | Pep 6 (81-120) |
|  | 5-GCTC*GTCGAC*TTAGCGTTGACTGATTTCA-3 |  |
| Pri7 | 5-GCAT*GAATTC*GACCAATATGCTGTTG-3 | Pep 7 (101-149) |
|  | 5-GAC*GTCGAC*TTAGTCAACCGTCTT-3 |  |
| Pri8 | 5-GAC*GAATTC*ATGGAGGTACGTGTGC-3 | Pep 8 (1-20) |
|  | 5-AAGC*GTCGAC*TTACAAATAGCTAGATGTTATTCC-3 |  |
| Pri9 | 5-TGAA*GAATTC*ACATCTAGCTATTTGAAGACT-3 | Pep 9 (16-35) |
|  | 5-GGGA*GTCGAC*TTACACAGTGTCCCAGGCTGTCTG-3 |  |
| Pri10 | 5-TGCA*GAATTC*GCCTGGGACACTGTGACTTTT-3 | Pep10 (31-50) |
|  | 5-AACA*GTCGAC*TTAACAATACGCATTGCCA-3 |  |
| Pri11 | 5-AATTCACATCTAGCTATTTGAAGACTCCTGCTTGCTAAG-3 | Pep 11 (16-25) |
|  | 5-TCGACTTAGCAAGCAGGAGTCTTCAAATAGCTAGATGTG-3 |  |
| Pri12 | 5-AATTCAAGACTCCTGCTTGCTGGAATGCACAGACATAAG-3 | Pep 12 (21-30) |
|  | 5-TCGACTTATGTCTGTGCATTCCAGCAAGCAGGAGTCTTG-3 |  |
| Pri13 | 5-AATTCTGGAATGCACAGACAGCCTGGGACACTGTGTAAG-3 | Pep 13 (26—35) |
|  | 5-TCGACTTACACAGTGTCCCAGGCTGTCTGTGCATTCCAG-3 |  |
| Pri14 | 5-AATTC GCCTGGGACACTGTGACTTTTCACGTCCCTGATGTAA  TTAGAGTTTAAG-3 | Pep 14 (31-45) |
|  | 5-TCGACTTA AACTCTAATTACATCAGGGACGTGAAAAGTCACA  GTGTCCCAGGCG-3 |  |
| Pri15 | 5-AATTC TGGGACACTGTGACTTTTCACGTCCCTGATGTAATTAG  AGTTGGC TAAG-3 | Pep 15 (32-46) |
|  | 5-TCGACTTAGCCAACTCTAATTACATCAGGGACGTGAAAAGTCA  CAGTGTCCCA G-3 |  |
| Pri16 | 5-AATTC GACACTGTGACTTTTCACGTCCCTGATGTAATTAGAGT  TGGCAAT TAAG-3 | Pep 16 (33-47) |
|  | 5-TCGACTTAATTGCCAACTCTAATTACATCAGGGACGTGAAAAG  TCACAGTGTC G-3 |  |
| Pri17 | 5-AATTC ACTGTGACTTTTCACGTCCCTGATGTAATTAGAGTTGG  CAATGCG TAAG-3 | Pep 17 (34-48) |
|  | 5-TCGACTTACGCATTGCCAACTCTAATTACATCAGGGACGTGAA  AAGTCACAGTG-3 |  |
| Pri18 | 5-AATTC GTGACTTTTCACGTCCCTGATGTAATTAGAGTTGGCAA  TGCGTAT TAAG-3 | Pep 18 (35-49) |
|  | 5-TGGACTTAATACGCATTGCCAACTCTAATTACATCAGGGACGT  GAAAAGTCACG-3 |  |
| Pri19 | 5-AATTC ACTTTTCACGTCCCTGATGTAATTAGAGTTGGCAATGC  GTATTGT TAAG-3 | Pep 19 (36-50) |
|  | 5-TCGACTTAACAATACGCATTGCCAACTCTAATTACATCAGGGA  CGTGAAAAGTG-3 |  |

Notes: All the primers were designed according to the S3 gene of ARV S1133 strain (GenBank accession number:U20642.1).The restriction endonuclease sites introduced in the Pri1 to Pri 10 pairs are in italics. In Pri11 to Pri 19 pairs, the 5’ end of the forward primer and 3’ of the reverse primer formed the sticky-ends of *EcoR*I restriction endonuclease sites. The same as *EcoR*I, the sticky-ends of the *Sal*I restriction endonuclease sites was formed by the 5’ end of reverse primer and 3’ of the forward primer. The positions where the peptides are in accordance with the published protein sequence of S1133 strain σB protein (UniProt accession number: E7AXT8).
