# Supplementary material for: Antigenic Analysis of Monoclonal Antibodies against Different Epitopes of σB Protein of Avian Reovirus
Source: PLoS One. 2013 Nov 27;8(11):e81533. doi: 10.1371/journal.pone.0081533 (PMC3842295; doi:10.1371/journal.pone.0081533)
Supplement: Table S2 — Virus sequences used for alignment and their accession numbers in GenBank. (DOCX) [file pone.0081533.s002.docx]

**Table S2. Virus sequences used for alignment and their accession numbers in GenBank**

| **Viruses** | **Host** | **Accession number** | **Viruses** | **Host** | **Accession number** |
| --- | --- | --- | --- | --- | --- |
| S1133 | Chicken | AF301472 | YH | Duck | DQ198856 |
| 919 | Chicken | AF208034 | NP03-CHN-2009 | Duck | GQ888710 |
| SI601 | Chicken | AF208037 | 89026 | Duck | AJ006476 |
| 176 | Chicken | AF059720 | 89030 | Duck | AJ243881 |
| 918 | Chicken | AF301473 | GZ-CHN-2007 | Duck | HM591301 |
| 1017 | Chicken | AF301474 | J18 | Duck | JX478268 |
| R2TW | Chicken | AF301472 | S12 | Duck | DQ643971 |
| 2408 | Chicken | AF208038 | YJL | Duck | DQ198855 |
| T6 | Chicken | AF208036 | TX99 | Turkey | AY444910. |
| 601G | Chicken | AY008384 | PEMS85 | Turkey | AY444913 |
| 1733 | Chicken | AF004856 | TX98 | Turkey | AY444911 |
| 750505 | Chicken | AF208035 | ATCCVR-818 | Turkey | AY444912 |
| 99G | Chicken | DQ415659 | NC-98 | Turkey | AF465799 |
| T98 | Chicken | EF030499 |  |  |  |
| B-98 | Chicken | EF030498 |  |  |  |
| C-98 | Chicken | EF030496 |  |  |  |
| G-98 | Chicken | EF030497 |  |  |  |
| OS161 | Chicken | AF301471 |  |  |  |
